# Chungkookjang with High Contents of Poly-γ-Glutamic Acid Improves Insulin Sensitizing Activity in Adipocytes and Neuronal Cells

**DOI:** 10.3390/nu10111588

**Published:** 2018-10-29

**Authors:** Seong-Yeop Jeong, Do Yeon Jeong, Da Sol Kim, Sunmin Park

**Affiliations:** 1 Department of R & D, Sunchang Research Center for Fermentation Microbes, Sunchang-Gun, Sunchang-yup 56048, Korea; khs8706@naver.com (S.-Y.J.); jdy2534@korea.kr (D.Y.J.); 2 Department of Food and Nutrition, Obesity/Diabetes Research Center, Hoseo University, Asan 31499, Korea; tpfptm14@daum.net

**Keywords:** Chungkookjang, glucose uptake, insulin secretion, insulin resistance, neuronal cell survival

## Abstract

We hypothesized that soybeans fermented with *Bacillus* spp. for 48 h (chungkookjang) would be rich in poly-γ-glutamate (γ-PGA) and would have greater efficacy for improving insulin sensitivity and insulin secretion in 3T3-L1 adipocytes, min6 cells, and PC12 neuronal cells. We screened 20 different strains of *B. subtillus* and *B. amyloliquefaciens* spp. for γ-polyglutamate (PGA) production and their anti-diabetic and anti-dementia activities in cell-based studies. Chungkookjang made with two *B. amyloliquefaciens* spp. (BA730 and BA731) were selected to increase the isoflavonoid and γ-PGA. Insulin-stimulated glucose uptake was higher in 3T3-L1 adipocytes given both chungkookjang extracts than in the cells given vehicle (control). The ethanol extract of BA731 (BA731-E) increased the uptake the most. Triglyceride accumulation decreased in BA731-E and BA731-W and the accumulation increased in BA730-W and BA730-E. The mRNA expression of fatty acid synthetase and acetyl CoA carboxylase was much lower in BA731-E and BA731-W and it was higher in BA730-W than the control. BA730-E and BA730-W also increased peroxisome proliferator-activated receptor (PPAR)-γ activity. Glucose-stimulated insulin secretion increased with the high dosage of BA730-W and BA730-E in insulinoma cells, compared to the control. Insulin contents and cell survival in high glucose media were higher in cells with both BA731-E and BA730-E. Triglyceride deposition and TNF-α mRNA expression were lower in BA731 than the control. The high-dosage treatment of BA730-E and BA731-E increased differentiated neuronal cell survival after treating amyloid-β(25-35) compared to the control. Brain-derived neurotrophic factor and ciliary neurotrophic factor, indices of neuronal cell proliferation, were higher in BA730 and BA731 than in the control. Tau expression was also reduced in BA731 more than the control and it was a similar level of the normal-control. In conclusion, BA730 increased PPAR-γ activity and BA731 enhanced insulin sensitivity in the brain and periphery. BA730 and BA731 prevented and alleviated the symptoms of type 2 diabetes and Alzheimer’s disease with different pathways.

## 1. Introduction

Insulin resistance is a common underlying factor in the etiology of type 2 diabetes (T2DM) and Alzheimer’s disease (AD) [1]. Insulin resistance is generated by the impairment of insulin signaling in different tissues. Although the etiology of insulin resistance is complicated, the increased production of pro-inflammatory cytokines and free radicals is associated with increased insulin resistance. Triglyceride accumulation in adipose tissues, especially in the abdomen, contribute to the release of various cytokines, such as tumor necrosis factor-α (TNF-α), interleukin-6 (IL-6), and monocyte chemoattractant protein-1 (MCP-1), into the circulation [2]. They are delivered into various tissues, including the brain, and contribute to the development of insulin resistance [2]. The deposition of amyloid-β is toxic to neurons, causing neuronal cell death and leading to the development of AD, as reported by *in vitro* and *in vivo* studies [3]. The amyloid-β plaque formation is known to be modulated by intracellular tau pathologies that are changed by brain insulin resistance, oxidative stress, and neuroinflammation [4]. The prevention of amyloid-β deposition in the early stages can protect against the development of AD in humans [3]. Increased insulin resistance impairs energy, glucose and lipid metabolism; and leads to the development of obesity, T2DM and dyslipidemia and it eventually impairs brain insulin sensitivity, which also contributes to AD [4]. These pathologies create a vicious cycle that exacerbates their symptoms. 

Although some insulin sensitizers have been developed, they have some adverse effects when taken for a long period [5]. People are interested in foods to prevent T2DM and AD. Some foods are reported to improve insulin sensitivity and chungkookjang is one of them [6,7]. Chungkookjang is a short-term soybean fermented foods without salt. It is traditionally fermented with rice straw at 40 °C for 2–3 days [6,8]. Chungkookjang is used to make soup with vegetables for meals on a daily basis and it is taken as it is like natto in Japan. The traditionally fermented chungkookjang mainly contains various *Bacillus* spp. The main *Bacilli* in the traditional chungkookjang include *Bacillus subtilus*, *Bacillus licheniformis*, and *Bacillus amyloqucences* [9]. Previous studies have demonstrated that chungkookjang fermented by *B. licheniformis* contains the highest anti-diabetic efficacy and it contains the most poly-γ-glutamic acid (γ-PGA) among the *Bacillus* spp. [9]. The consumption of chungkookjang fermented with *B. licheniformis* potentiates glucose-stimulated insulin secretion and also improved insulin sensitivity in partially pancreatectomized rats, a lean type 2 diabetic animal model [7,10]. In addition, the intake of chungkookjang fermented with *B. licheniformis* enhances glycemic control in obese mice and decreases body fat mass [11]. However, *B. licheniformis* is not registered as a food ingredient by the Korea Food & Drug Administration due to possible toxicity. Thus, we are interested in exploring other *Bacilli* that make γ-PGA during the fermentation of soybeans and to investigate their anti-diabetes and anti-dementia efficacies. 

Chungkookjang contains phytochemicals, including isoflavonoids, and γ-PGA, that may work as carriers to improve insulin resistance by the phytochemicals in chungkookjang. Glycated isoflavonoids such as daidzin and genistin in soybeans have estrogenic activities to modulate energy, glucose and lipid metabolism. Fermentation of soybeans converts glycated isoflavones into aglycated ones including daidzein and genistein. Aglycated isoflavones have higher functions than glycated ones. In addition, γ-PGA is produced during soybean fermentation. γ-PGA has been used as a nanopolymeric oral drug carrier to deliver undigested complex drugs, such as insulin, to the circulatory system [12], but it has not been examined for use as a hypoglycemic agent. The components that make up the complexes with γ-PGA have insulin sensitizing activities, but without being digested in the gastrointestinal tract [13]. γ-PGA is produced by certain *Bacillus* spp. more than other *Bacilli* during the fermentation of soybeans. Thus, we hypothesized that chungkookjang rich in γ-PGA improved insulin sensitivity and insulin secretion and reduced acetate in cell-based experiments. We tested the hypothesis in 3T3-L1 adipocytes, Min6 cells, and PC12 cells. 

## 2. Materials and Methods

### 2.1. Preparation of Bacillus spp.

*B. amyloliquefaciens* SRCM100730 and SRCM100731 were selected from among the strains tested and used for producing chungkookjang. Each *Bacillus* spp. was cultivated in Luria-Bertani broth at 37 °C with shaking (128 rpm, Jeio Tech, Daejeon, Korea) to increase the number of *Bacilli* and it was added into steamed soybeans to ferment them immediately after the culture [9]. 

### 2.2. Preparation of Standardized Chungkookjang

Soybeans were steamed at 121 °C for 1 h, and cooled to 40 °C. The steamed soybeans were inoculated with either *B. amyloliquefaciens* SRCM100730 or SRCM100731 at concentrations of 10^7^–10^8^ CFU∙mL^−1^ and incubated at 42 °C for 48 h. Previous and preliminary studies showed that a fermentation temperature of 42 °C is optimal for preventing fungal growth while permitting rapid growth of the fermentative microorganisms [9]. Its anti-diabetic activity was optimal after 48 h incubation [8]. As a control, cooked soybeans were incubated without inoculation with *Bacillus* and it was extracted with 70% ethanol and it was lysophilized (CSB-E). Since the CSB-E contained more isoflavonoids, it was used as a control. After making chungkookjang fermented with *B. amyloliquefaciens* SRCM100730 or SRCM100731 (BA730 and BA731), each was lysophilized and extracted with 70% ethanol (BA730-E or BA731-E) or 100% water (BA730-W or BA731-W) by shaking for 24 h at 25 °C, and then precipitates were removed by centrifuging at 8000× *g* for 30 min. The supernatants were freeze-dried. 

### 2.3. Isoflavonoid Contents

The 70% Ethanol extract of chungkookjang was used for measuring isoflavonoids. HPLC was performed using a JASCO liquid chromatography system (JASCO, Tokyo, Japan) equipped with an autoinjector and a UV detector. The contents of isoflavonoids were analyzed with an YMC ODS-AM column (4.6 × 250 nm, 5 µm, Waters). The mobile phase was 0.1% acetic acid aqueous solution (A) and 0.1% acetic acid in acetonitrile (B). The gradients used were as follows: 0 min, A:B = 88:12 (*v*/*v*); 18 min, A:B = 78:22; 28 min, A:B = 72:28; 35 min, A:B = 62:38; 48 min, A:B = 52:48; 54 min, A:B = 32:68; 58 min, A:B = 0:100. The mobile phase flow rate was 1.0 mL/min under the following conditions: column temperature, 35 °C; injection volume, 20 μL; and UV detection at 285 nm [14]. Standards used to identify compounds were daidzin, genistin, glycetin, daidzein, genistein, malonyl daidzein, malonyl genistein, acetyl daidzein, acetyl genistein and acetyl glycitin (0–100 μg/mL; Sigma Co., St. Louis, MO, USA).

### 2.4. γ-PGA Contents

γ-PGA is a viscous polymer of glutamic acids. It is produced in cooked soybeans during fermentation, and the amount produced is dependent on the *Bacillus* spp. The length of the γ-PGA on the surface of the chungkookjang was measured as an indicator of the γ-PGA production. 

### 2.5. Analysis of Free Amino Acid Contents

Water extracts of the chungkookjang samples were used for measuring the contents of free amino acids. The extracts were dissolved into water and centrifuged at 3000 rpm for 10 min and the supernatants were separated. 5% (*v*/*w*) tricarboxylic acid solution was added into the supernatants and the well-mixed solutions were left to stand at 4 °C for 1 h. After centrifugation, the isolated supernatants were mixed with 0.02 N HCL and filtered through a 0.22 μm membrane. The filtrates were injected into a high speed free amino acid analyzer (Hitachi L-8900; Minato-ku, Tokyo, Japan) to assess the contents of the free amino acids. The conditions used in the free amino acid analyzer were as follows: Mobile phase buffer set, pH-SET KANTO; separation column, Hitachi 4.6 × 60 mm and ammonia filtering column, Hitachi 4.6 × 40 mm; UV/Vis detector (440–570 nm); buffer flow rate, 0.40 mL/min; ninhydrin flow rate, 0.35 mL/min; temperature, 50 °C; and injection volume, 20 μL. 

### 2.6. Cell Culture

3T3-L1 fibroblasts and PC12 cells were purchased from ATCC (Manassas, VA, USA) and Min6 cells were generously provided by Dr. Miyazaki (Osaka University, Osaka, Japan). They were grown in high-glucose Dulbecco’s Minimal Essential Medium (DMEM; Invitrogen, Carlsbad, CA, USA) containing 10% fetal bovine serum (FBS), and 1% penicillin/streptomycin mixture at 37 °C with 5% CO_2_. 3T3-L1 fibroblasts were differentiated into preadipocytes with differentiation inducers—1 mg∙mL^−1^ of insulin (Sigma Co., St. Loise, MO, USA), 50 μM of dexamethasone (Sigma Co., St. Loise, MO, USA), and 0.8 mM of isobutyl methyl xanthine (Sigma Co., St. Loise, MO, USA)—for 4 days and then continuously differentiated into adipocytes in high-glucose DMEM containing 10% FBS. PC12 cells were differentiated with nerve growth factor (NGF) for 2 days. Differentiated PC12 cells were treated with 10 μM amyloid-β (25–35) (Sigma Co., St. Loise, MO, USA) to induce neuronal cell apoptosis for two days.

### 2.7. Cell Viability

Cell viability was measured in the 3T3-L1 adipocytes, Min6 insulinoma cells and differentiated and amyloid-β administered PC12 neuronal cells treated with either vehicle (DMSO or water) or designated concentrations of cooked soybeans (CSB, control), different chungkookjang extracts or metformin using an 3-(4,5-dimethylthiazol-2-yl)-2,5-diphenyltetrazolium bromide (MTT) assay with an Aureon plate reader (Aureon Biosystems, Vienna, Austria). 

### 2.8. Insulin-Stimulated Glucose Uptake

At 9–12 days after the initiation of differentiation, the adipocytes and differentiated PC12 cells were used for determining insulin-stimulated glucose uptake by seeding into 24-well plates at 4 × 10^4^ cells per well in high-glucose DMEM containing 10% FBS for 6 h [14]. The medium was then switched to low-glucose DMEM containing 0.3% bovine serum albumin (BSA) and the ethanol extracts of CSB or 0.5 or 5 μg∙mL^−1^ each of the methanol and water extracts (BA730-W, BA730-E, BA730-W and BA731-E) and incubated at 37 °C for 16 h. The medium was switched to a Krebs–Ringer–Hepes (KRH) buffer containing either 1.0 ng/mL insulin with CSB-E, or 0.5 or 5 μg∙L^−1^ extracts and further incubated at 37 °C for 30 min. Insulin-stimulated glucose uptake was then measured using a colorimetric glucose uptake assay kit (Abcam, Cambridge, MA, USA). As positive controls, rosiglitazone (0.5 or 2 μM) plus 1.0 ng/mL insulin and 50 ng/mL insulin were used. Non-specific glucose uptake was measured in cells treated with DMSO or extracts without insulin. 

### 2.9. Triglyceride Accumulation in 3T3-L1 Adipocytes and Min6 Insulinoma Cells

Vehicle (DMSO) or 5 μg∙mL^−1^ 70% ethanol extracts of CSB and chungkookjangs were added into media with the differentiation inducers for 4 days during the differentiation of 3T3-L1 fibroblasts, and then the cells were treated with CSB-E (control) or extracts (BA730-W, BA730-E, BA730-W and BA731-E) without differentiation inducers for 6 additional days. Triglyceride contents of treated cells were determined after 6 days of incubation with the extracts. The cells were harvested with a lysis buffer without glycerol and the triglyceride contents in the cells were measured using a Trinder kit (Young Dong Pharmaceutical Co., Seoul, Korea) as previously described [14]. The results were expressed as the percentage of change in triglyceride concentration from the baseline (vehicle treatment). 

### 2.10. Peroxisome Proliferator-Activated Receptor-Gamma (PPAR-γ) Agonist Activity

Human embryonic kidney (HEK) 293 cells were transiently transfected with a PPAR-*γ* responding element (PPRE)-luciferase construct (firefly pGL3-DR-1-luciferase; 0.12 μg DNA∙well^−1^), pSV-SPORT-PPAR-γ expression vector (0.12 μg DNA∙well^−1^) and pSV-SPORT-retinoid X receptor-α vector (0.08 μg DNA∙well^−1^) with a Lipofectamine PLUS reagent (Invitrogen, Carlsbad, CA, USA) according to the manufacturer’s protocol. PPAR-γ activity was measured as described in previous studies [9]. Briefly, for an assessment of transfection efficiency, renilla phRL-TK vector (10 ng DNA∙well^−1^, Promega, Madison, WI, USA) was also transfected. After 2 h of transfection, 5 μg∙mL^−1^ extracts of CSB-E (control) or different chungkookjang extracts (BA730-W, BA730-E, BA730-W or BA731-E) were added into media for 40 h and the media was changed to serum-free DMEM containing 0.1% BSA, which also contained the respective extracts, and held for 12 h [9]. Cell lysates were assayed for both firefly (PPRE-luciferase) and renilla luciferase activities using the Dual-Luciferase Reporter Assay System (Promega) and an Aureon PhL luminometer (Aureon Biosystems, Vienna, Austria). Ratios of firefly luciferase activity and renilla luciferase activity were calculated for results.

### 2.11. Glucose-Stimulated Insulin Secretion

Min6 cells were grown as previously described by Park et al. [15] in a 24 well plate at 6 × 10^4^ cells per well with high glucose DMEM containing 0.3% BSA and either CSB-E (control) or 5 μg∙mL^−1^ different chungkookjangs (BA730-W, BA730-E, BA730-W or BA731-E) for 16 h. Exendin-4 (0.5 or 2.5 nM; Sigma Co., St. Louis, MO, USA) treated cells were used as a positive control. After washing the cells with PBS, the Min6 cells were treated with 0.5 or 5 μg∙mL^−1^ respective extracts in low (2 mM) or high glucose (20 mM) KRH buffer containing 20 mM Hepes pH 7.4 and 5 mg∙mL^−1^ BSA for 30 min. Insulin concentrations in supernatants from all wells were measured using a ELISA kit (Crystal Chem, Elk Grove Village, IL, USA). 

Min6 cells were grown in high glucose (20 mM) DMEM media for 48 h with CSB-E, ethanol or water extracts of different chungkookjangs. After harvesting the cells, the contents of insulin and triglyceride in the Min6 insulinoma cells were measured. After making cDNA from the total RNA of the Min6 cells treated with the chungkookjang, TNF-α mRNA expression was measured by real-time PCR.

### 2.12. Gene Expression

After the treatment with 5 μg∙min^−1^ of control, 70% ethanol and water extracts of BA730 and BA731 chungkookjangs in 3T3-L1 adipocytes and PC12 cells, cDNA from both cell lines was generated. The mRNA expression of genes of interest was measured by real-time PCR. Total RNA was isolated from the adipose tissues using a monophasic solution of phenol and guanidine isothiocyanate (Trizol reagent, Gibco-BRL, Rockville, MD, USA), followed by extraction and precipitation with isopropyl alcohol. The cDNA was synthesized from equal amounts of total RNA with superscript III reverse transcriptase, and polymerase chain reaction (PCR) was performed with high fidelity Taq DNA polymerase [15]. Equal amounts of cDNA were mixed with sybergreen mix and they were analyzed using a real-time PCR machine (BioRad Laboratories, Hercules, CA, USA). The expression level of the gene of interest was corrected for that of the house keeping gene, glyceraldehydes 3-phosphate dehydrogenase. The primers of fatty acid synthetase (FAS) and acetyl CoA carboxylase-1 (ACC1), tumor necrosis factor (TNF)-α, brain-derived neurotrophic factor (BDNF) and ciliary neurotrophic factor (CNTF) and tau were used. The sequences and characteristics of the primers were provided in Table 1. Their relative gene expression was quantitated using the comparative cycle of threshold (CT) method (2^−ΔΔCT^ method) as previously described by Livak and Schmittgen [16]. 

### 2.13. Immunoblot Analysis

Differentiated PC12 cells with NGF were treated with the vehicle, BA730-E, BA730-W, BA731-E, and BA731-W for 24 h and the cells were administered with 2 nM insulin for 30 min. The cells were harvested with lysate buffer and immunoblot analysis was conducted. Antibodies used for the immunoblot analysis were protein kinase B (PKB or Akt), phosphorylated PKB^Ser473^, glycogen synthase kinase (GSK) 3β, phosphorylated GSK-3β^ser9^, and β-actin (Cell Signaling Technology, Danvers, MA, USA). The intensity of protein expression was analyzed by Imagequant TL (GE Healthcare Bio-Sciences, Pittsburgh, PA, USA).

### 2.14. Statistical Analyses

All results are expressed as means ± standard deviation (SD). Statistical analysis was performed using SAS version 9.1 (SAS Institute, Cary, NC, USA). Significant differences in isoflavone contents and peptides were determined in the control (70% ethanol extract of cooked soybean) and 70% ethanol and water extracts of BA730 and BA731 by one-way ANOVA. Two-way ANOVA was used to determine the effects of *Bacilli* spp. and extraction solvents. Significant differences in the main effects among the groups were identified by Tukey’s test. The positive control (rosiglitazone or exendin-4 treatment) and control treatment was compared using a two-sample *t*-test. *p* < 0.05 was considered statistically significant. 

## 3. Results

### 3.1. Isoflavone Contents of Water Extracts of Chungkookjang

Isoflavonoid glycosides and malonyl and acetyl isoflavoids were decreased in BA730 and BA731, whereas BA731 decreased daidzin and genistin compared to that made with BA730. Total isoflavones were similar between soybeans and chungkookjang with BA731 but they were lower in BA730 than that with BA731 (Table 2). However, isoflavoinoid aglycones were much higher in BA730 and BA731 than soybeans and they were lower in BA730 than that in BA731 (Table 2). 

### 3.2. Amino Acid and γ-PGA Contents of Water Extracts of Chungkookjang

**Figure 1:**
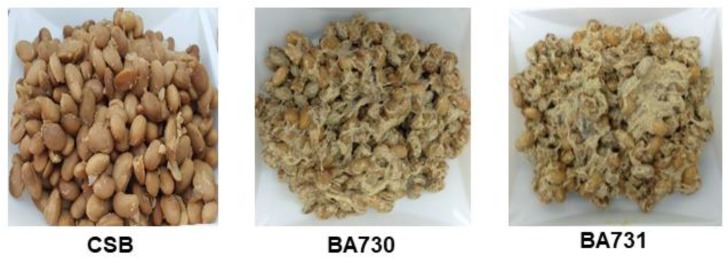
Photo of cooked soybeans (CSB), chungkookjang made with *B. amyloliquefaciens* SRCM100730 (BA730), chungkookjang made with *B. amyloliquefaciens* SRCM100731 (BA731).

Most amino acids increased in BA730 and BA731 compared to CSB (Table 3). The amount of free amino acid increased in chungkookjang due to the degradation of protein by bacteria. However, glutamate did not exist in chungkookjang, which indicated that glutamate had been used. The metabolites of amino acids such as urea, α-aminoadipic acid, citrulline, α-, β-, γ-aminoisobutyric acid, ethanolamine, ammonia, and ornithine were elevated in both chungkookjang extracts with some variation (Table 3). The contents of γ-PGA were higher in both chungkookjangs (BA730 and BA731) (Table 4; Figure 1). 

### 3.3. Bacterial Survival Rates in Low Acidity and High Concentration of Bile Salts

BA731 survived better than bacteria BA730 but the survival rates of BA730 was much higher in bile salts than BA731 (Table 4). 

### 3.4. Insulin-Stimulated Glucose Uptake in 3T3-L1 Adipocytes

**Figure 2:**
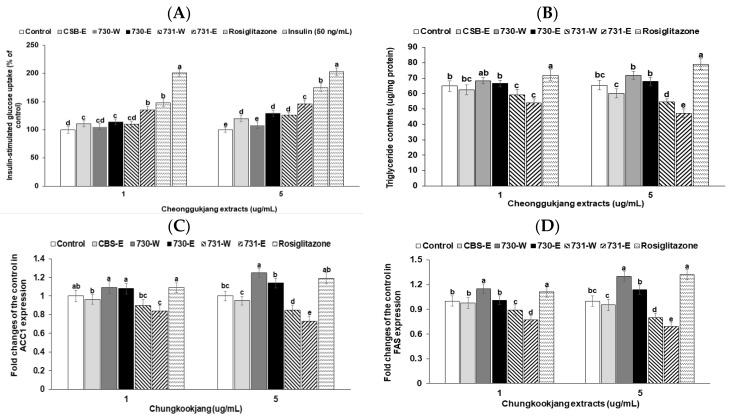
Insulin-stimulated glucose uptake, triglyceride deposition and expression of genes related to fatty acid synthesis in 3T3-L1 adipocytes. 3T3-L1 adipocytes were pre-treated with the 48 h-treatment of the ethanol (-E) and water (-W) extracts (1 and 5 μg/mL) of chungkookjang fermented with *B.* *amyloliquefaciens* SRCM100730 (BA730) and SRCM10073 (BA731). (**A**) Insulin-stimulated glucose uptake was measured with low dosage (1 ng/mL) of insulin. Insulin treatment of 50 ng/mL was considered as normal-control. (**B**) Triacylglycerol contents were determined in 3T3-L1 adipocytes after 9–10 days of differentiating from 3T3-L1 fibroblast with differentiation inducers and vehicle, 1 or 5 μg∙mL^−1^ of ethanol (-E) or water (-W) extracts of BA730 and BA731. (**C**,**D**) The fold changes of mRNA levels of genes associated with fatty acid metabolism (ACC1 and FAS) from the control were calculated by the 2^−ΔΔCT^ method after conducting real-time PCR. The control and positive control were used as ethanol extracts of cooked soybeans and rosiglitazone (0.5 or 2 μM), respectively. Values are means ± SD (*n* = 7). ^a,b,c,d,e^ Different letters above the bars indicate significant differences among the groups by Tukey’s test at *p* < 0.05.

Non-specific glucose uptake without insulin addition was not different between vehicle (control) and chungkookjang water and ethanol extracts in 3T3-L1 adipocytes. Insulin treatment increased glucose uptake and over 50 ng/mL insulin resulted in a plateau of glucose uptake (Figure 2A). In order to check the chungkookjang extracts as insulin sensitizers a low concentration of insulin (1 ng/mL) was added into each treatment of chungkookjang extracts in 3T3-L1 adipocytes. CSB-E elevated insulin-stimulated glucose uptake more than the control and BA730-E elevated the glucose uptake as much as CSB-E. However, the increase was not big. The low and high dosages of BA731-W and BA731-E markedly increased insulin-stimulated glucose uptake compared to the control. BA731-E increased glucose uptake the most among the chungkookjang extracts (Figure 2A). Only BA731-E (5 μg/mL) exhibited a similar increase of insulin-stimulated glucose uptake as with rosiglitazone in 3T3-L1 adipocytes (Figure 2A). However, the increase in the high dosage of 731-E and rosiglitazone was not as much as 10 nM insulin treatment (Figure 2A).

### 3.5. Triglyceride Accumulation and Expression of Genes Related to Fatty Acid Synthesis in 3T3-L1 Adipocytes

*Bacillus* spp. altered differentiation of 3T3-L1 fibroblasts and accumulation of triglyceride in 3T3-L1 adipocytes (Figure 2B). The high dosage of BA730-W and BA730-E bacteria slightly increased triglyceride accumulation compared to the control, whereas BA731-E decreased triglyceride accumulation (Figure 2B). However, rosiglitazone increased the triglyceride storage compared to the control (Figure 2B). These results indicated that BA730 and BA731 had different pathways to alter glucose metabolism and chungkookjang made with BA730 might have a similar pathway to rosiglitazone. Thus, two *Bacillus* spp. made different metabolites to influence the modulation of triglyceride metabolism. 

FAS and ACC1 are known regulators of fatty acid synthesis. High dosage of BA730-W had a higher fold change of ACC1 expression from the control but BA731-W and BA731-E had a lower fold change of ACC1 expression from the control in 3T3-L1 adipocytes (Figure 2C). FAS expression exhibited a similar pattern as with ACC1 expression but the fold change of FAS expression tended to have a higher effect. Therefore (Figure 2D), BA730-W exhibited the trend to increase the expression of genes related to fatty acid synthesis but BA731-W and BA731-E decreased their expression.

### 3.6. PPAR-γ Activity

**Figure 3:**
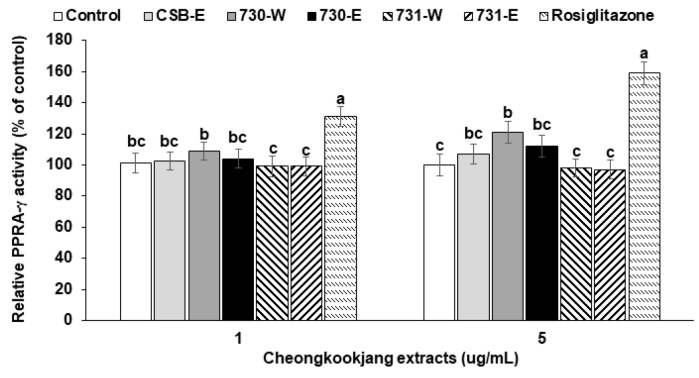
Peroxisome proliferator-activated receptor (PPAR)-γ activation in human embryonic kidney 293 cells. 3T3-L1 adipocytes were pre-treated with the 48 h-treatment of the ethanol (-E) and water (-W) extracts (1 and 5 μg/mL) of cooked soybeans (CSB), chungkookjang fermented with *B. amyloliquefaciens* SRCM100730 (BA730) and SRCM100731 (BA731) in human embryonic kidney 293 cells. PPAR-γ activity was measured by luciferase activity after transfection of PPAR response element (PPRE)-luciferase construct, pSV-SPORT-PPAR-γ expression vector, pSV-SPORT-retinoid X receptor (RXR)-α vector, and renilla phRL-TK vector. The control and positive control used was the ethanol extracts of CSB and rosiglitazone (0.5 or 2 μM), respectively. Values are means ± SD (*n* = 7). ^a,b,c^ Different letters above the bars indicate significant differences among the groups by Tukey’s test at *p* < 0.05.

High dosage of BA730-W slightly increased PPAR-γ activity in HEK 293 cells transfected PPAR-γ expression related genes compared to the control and CSB-E but the increase was much less than with 2 μM rosiglitazone, a PPAR-γ agonist (Figure 3). BA731-E and BA731-W did not alter PPAR-γ activity (Figure 3). 2 μM rosiglitazone is about 715 pg/mL, which is similar to Cmax of serum rosiglitazone following the 8 mg single dose rosiglitazone intake [17]. A high dosage of 730-W was shown to activate PPAR-γ.

### 3.7. Glucose-Stimulated Insulin Secretion and Cell Viability in Min6 Insulinoma Cells

**Figure 4:**
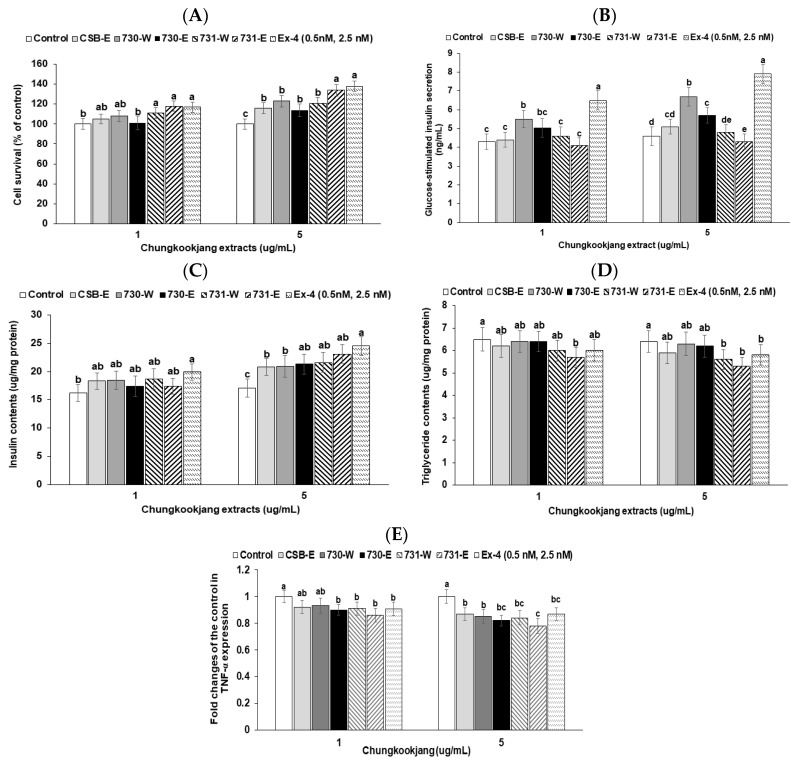
Glucose-stimulated insulin secretion, cell survival, insulin and triglyceride contents and TNF-α expression in insulinoma Min6 cells. Cell survival (**A**) was measured after 24 h treatment of vehicle or 5 μg·mL^−1^ of 70% ethanol (-E) or water extracts (-W) of chungkookjang fermented with *B. amyloliquefaciens* SRCM100730 (BA730) and SRCM100731 (BA731). Glucose-stimulated insulin secretion (**B**) measured in high glucose (20 mM) Krebs–Ringer–Hepes buffer for 30 min after treatment with vehicle or 5 μg·mL^−1^ of BA730-E, BA730-W, BA731-E and BA731-W. The contents of insulin (**C**) and triglyceride (**D**) in the Min6 insulinoma cells measured in high glucose (20 mM) DMEM media for 48 h with CSB-E, ethanol or water extracts of different chungkookjangs. TNF-α mRNA expression (**E**) in the Min6 cells treated with the chungkookjang was determined by real-time PCR. The positive control was exendin-4 (Ex-4). Values are means ± SD (*n* = 7). ^a,b,c,d,e^ Different letters above the bars indicate significant differences among the groups by Tukey’s test at *p* < 0.05.

The cell survival by β-cell proliferation was elevated with BA730-W, BA730-E, BA731-W, and BA731-E in comparison to the control (Figure 4A). Cell survival was higher with BA731 as much as the positive-control (exendin-4) (Figure 4A). Insulin secretion in Min6 insulinoma cells was 4.7 ± 0.6-fold higher in the high glucose (20 mM) KRH buffer without chungkookjang extracts than in the low glucose (2 mM) KRH buffer. Chungkookjang extracts did not change insulin secretion in low glucose KRH buffer. BA730-W and BA730-E increased glucose-stimulated insulin secretion compared to the control and CSB-E and the increase was higher in the high-dosage treatment than the low-dosage treatment (Figure 4B). The BA730-W had a higher increase of glucose-stimulated insulin secretion than the BA730-E (Figure 4B). 

To sustain glucose-stimulated insulin secretion, high insulin contents in the cells needed to be maintained. The β-cell mass needs to also be increased. Insulin contents were higher in the Min6 cell treated with water and ethanol extracts of BA730 and BA731 than in the control (Figure 4C). The β-cell function is reported to be associated with triglyceride contents. Triglyceride contents in the cells were lower in BA731-W and BA731-E than the control and CSB-E (Figure 4D). 

TNF-α expression, an index of inflammation, was tended to be down-regulated with water and ethanol extracts of all chungkookjang compared to the control (Figure 4E). A high dosage of BA731-E decreased the fold changes of TNF-α expression from the control in Min6 cells (Figure 4E). 

### 3.8. Differentiated Neuronal Cell Survival

**Figure 5:**
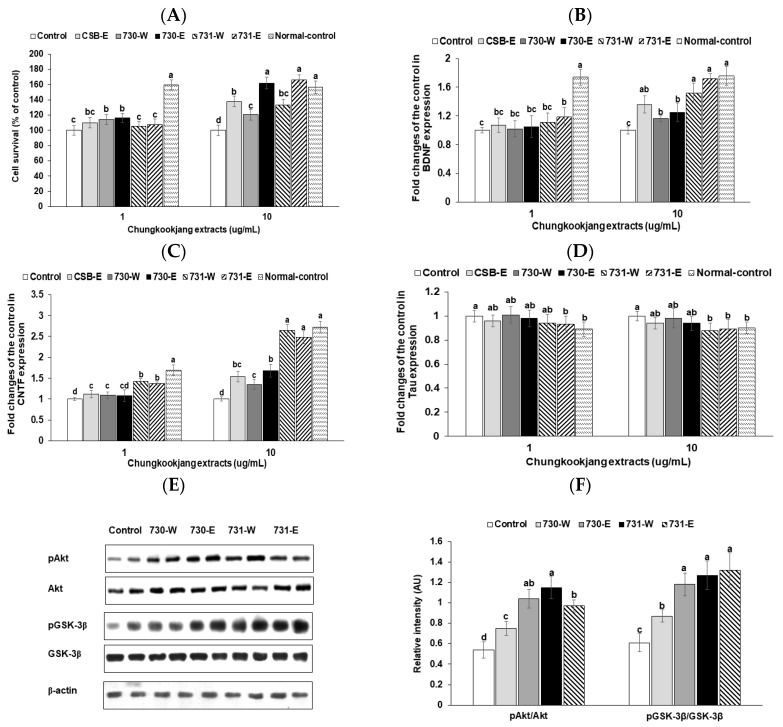
Cell survival and mRNA expression of genes associated with neurogenesis in differentiated PC12 cells. PC12 cells were differentiated with nerve growth factor (NGF) for 2 days and differentiated PC12 cells were administered with amyloid-β (25–35) for additional 2 days. The PC12 cells had treatments with a vehicle or 5 μg∙mL^−1^ of 70% ethanol (-E) or water extracts (-W) of chungkookjang fermented with *B.* *amyloliquefaciens* SRCM100730 (BA730) and SRCM100731 (BA731). Cell survival (**A**) was measured after 24 h treatment. The cDNA was generated from the cells and the fold changes of mRNA levels of BDNF (**B**), CNTF (**C**), and tau (**D**) from the control were calculated by the 2^−ΔΔCT^ method after conducting real-time PCR. The phosphorylation of Akt and GSK-3β was conducted by immunoblooting and intensity of the blots was measured (**E**). Immunoblot assay was performed with high dosage of assigned chungkookjang extracts. The relative intensity of phosphorylation of Akt and GSK-3β was given (**F**). Values are means ± SD (*n* = 7). ^a,b,c,d^ Different letters above the bars indicate significant differences among the groups by Tukey’s test at *p* < 0.05.

Cell survival of differentiated PC12 cells with NGF-1 was reduced by 1.54 folds with amyloid-β (25–35) treatment in the control group (Figure 5A). Cell apoptosis was prevented by water and ethanol extracts of chungkookjang at low dosages. High dosage of all chungkookjang extracts increased cell survival under amyloid-β (25–35) treatment, compared to the control. BA730-E and BA731-E had greater efficacy for cell survival than BA730-W and BA731-W (Figure 5A). The improvement was similar to the normal-control in both BA730-E and BA731-E.

BDNF mRNA expression increased in the high dosage of both chungkookjang extracts. BA731-E increased BDNF expression as much as the normal-control (Figure 5B). CNTF mRNA expression was also elevated with both chungkookjang extracts but BA731-W and BA731-E increased CNTF mRNA expression more than those with BA730-W and BA730-E and the increase was as much as normal-control (Figure 5C). The mRNA expression of tau, an index of amyloid deposition, was lowered only in BA731-E and BA731-W to less than the control and their expressions were similar to the normal-control (Figure 5D).

### 3.9. Insulin Signaling Pathways

The phosphorylation of Akt was increased with a high dosage of chungkookjang extracts in differentiated PC12 cells compared to the control (Figure 5E). BA731-W elevated the phosphorylation of Akt the most among the groups. The phosphorylation GSK-3β was also increased with chungkookjang extracts in comparison to the control (Figure 5E). The phosphorylation of GSK-3β was elevated by BA730-E, BA731-W, and BA731-E more than the control. 

## 4. Discussion

Insulin resistance is a common manifestation of metabolic syndrome, which is also linked to dyslipidemia, hyperglycemia, hypertension, and AD [3]. The etiologies of these metabolic diseases are interrelated. When insulin resistance increases, the other metabolic diseases are induced. The occurrence of insulin resistance increases with aging and when insulin secretion capacity cannot increase enough to compensate for insulin resistance, aged people become susceptible to T2DM [18]. In addition, increased systemic insulin resistance contributes to the exacerbation of brain insulin resistance, which can contribute to the development of AD [1]. Although some foods are known to reduce insulin resistance, more foods need to be explored to alleviate insulin resistance. The major *bacillus* spp. in the traditional Korean fermented soybean food, chungkookjang, are *B. subtillus*, *B. licheniformis* and *B. amyloliquefaciens*. Each *Bacillus* species has different strains of bacteria, and they secrete different enzymes that degrade the soybeans. Previous studies have shown that chungkookjang fermented with *B. licheniformis* produces γ-PGA and it improves glucose metabolism and cognitive function better than traditionally fermented chungkookjang [9]. However, *B. licheniformis* has not been registered as an edible bacteria by the Korea Food & Drug Administration. In our preliminary study, we explored 20 different types of *B. subtilus* and *B. amyloliquefaciens* to determine which can improve glucose metabolism and neuronal cell survival. However, water extracts of chungkookjang contained more free amino acids and small peptides, and ethanol extracts of chungkookjang included more isoflavonoids. The chungkookjang extracts with different solvents contained somewhat different functional compounds. 

In the present study, we hypothesized that chungkookjang fermented with Bacillus to produce higher concentrations of γ-PGA would improve insulin sensitivity and insulin secretion in 3T3-L1 adipocytes, Min6 cells and PC12 cells. Two types of *B. amyloliquefaciens* spp. were selected due to a high production of γ-PGA. γ-PGA has reported to be produced by *B. licheniformis* and *B. subtilus* from glutamate in specific media [19] and they also produce γ-PGA in soybean fermentation (chungkookjang). Some studies have been conducted to increase γ-PGA production by different methods from *B. amyloliquefaciens* [20]. However, *B. amyloliquefaciens* is reported to produce γ-PGA but it is dependent on *B. amyloliquefaciens* spp. γ-PGA inhibits free fatty acid release by 50% in isolated rat adipocytes treated with epinephrine in comparison to the control [21], suggesting that γ-PGA has insulin mimicking activity. The consumption of γ-PGA also ameliorates hyperglycemia and metabolic syndrome in type 2 diabetic mice [22]. Thus, γ-PGA has an anti-diabetic activity. 

Although BA730 and BA731 both had increased γ-PGA, they may have different pathways to improve anti-diabetic activity by modulating insulin-stimulated glucose uptake and insulin secretion and to increase neuronal cell survival. The differences could be associated with different amounts of isoflavone aglycans. Furthermore, chungkookjang made with BA731 protected against damage by amyloid-β in neuronal cells better than that made with BA730. Thus, chungkookjang made with BA730 and BA731 have different anti-diabetic and anti-AD activities. Even though BA730 and BA731 are similar in their production of γ-PGA, they showed different contents of isoflavonoids and free amino acids that may be associated with different enzyme activities. *B. amyloliquefaciens* has been mainly studied for anti-microbial and anti-infection properties, and for biocontrol of plant-growth-promoting bacteria [23,24]. Furthermore, one of the *B. amyloliquefaciens* spp. produces 1-deoxynojirimycin, an effective α-glucosidase inhibitor to prevent carbohydrate digestion, and it can be used to make anti-diabetic product [25]. 

The different mechanism of action with different chungkookjangs might be associated with different levels of isoflavonoids, free amino acids and peptides. In the present study, BA730 and BA731 had anti-diabetic activity, but the action mechanism was different. BA730-W increased PPAR-γ activity and glucose-stimulated insulin secretion whereas the BA731-E decreased triglyceride accumulation by decreasing mRNA expression of FAS. The different action mechanisms to modulate insulin resistance and insulin secretion are also influenced by the changes in the contents of isoflavonoid glycans and aglycans, amino acids, peptides and soyasaponins. BA731 had more isoflavone aglycans and free amino acids than BA730 although γ-PGA production was similar in both chungkookjangs. Chungkookjang also has increased daidzein and genistein, isoflavone aglycons, which are more effective for regulating glucose metabolism than daidzin and genistin. 

An important feature of insulin resistance is the attenuation of insulin-stimulated glucose uptake in adipocytes and increased hepatic glucose output. In addition, the attenuation of insulin signaling in the hippocampus is associated with memory impairment. When hippocampal insulin resistance increases, the deposition of amyloid-β is elevated by increasing tau phosphorylation [3]. Insulin sensitizers alleviate insulin resistance in the periphery and brain to attenuate the clinical symptoms of T2DM and AD, respectively. In the present study, BA730 and BA731 exhibited insulin sensitizing activities mediated by different pathways. BA731 protected against neuronal cell death by amyloid-β, a toxic agent to neuronal cells, through improving insulin signaling to reduce tau expression. 

In the present study, the effects of chungkookjang extracts on insulin sensitivity, insulin secretion, and neuronal cell viability were assessed *in vitro* that is the potential limitation. It is uncertain that the results of *in vitro* studies can be applied to the animal and human studies. The previous studies have shown that the extracts of chungkookjang fermented with *B. licheniformis* or have improved glucose-stimulated insulin secretion in Min6 cells and insulin sensitivity in 3T3-L1 adipocytes [9]. The chungkookjang has also alleviated diabetic symptoms in partial pancreatectomized rats, a type 2 diabetic animal model [7,10] and it has also reduced memory deficit in amyloid-β infused rats, an AD animal model [6]. Furthermore, 12-week treatment of chungkookjang extract has reported to reduce body fat and waist circumferences and increase lean body mass in overweight/obese women and it has improved lipid profiles in all obese subjects in a double-blinded, randomized, crossover, placebo-controlled clinical trial [26]. Thus, the results of *in vitro* studies can be applied to animal and human studies. 

## 5. Conclusions

BA730 and BA731 prevent insulin resistance in cells representing the brain and periphery but they showed different efficacies, although both chungkookjang had rich γ-PGA contents. The major pathway of BA731-W and BA731-E was associated with the improvement of insulin signaling and BA730-W acted as a PPAR-γ agonist and increased glucose-stimulated insulin secretion. We suggest that chungkookjang fermented with BA731 and/or BA730 had the potential to prevent and alleviate the symptoms of T2DM and AD by different pathways.

## Figures and Tables

**Table 1:** The sequences and characteristics of primers used for real-time polymerase chain reaction (PCR) analysis.

Gene		Primer		Melting Temperature (°C)	Product Size (bp)	Gene Bank Number
Rat	BDNF	F	ATGCCGAACTACCCAATCGT	56	79	NM_001270638.1
		R	TGTACATACACAGGAAGTGTC			
	CNTF	F	CTCTGTAGCCGCTCTATCTG	59	105	NM_170786
		R	GGTACACCATCCACTGAGTC			
	Tau	F	AAGACAGACCATGGAGCAGAAATC	62	118	NM_017212.2
		R	CGGCTAACGTGGCAAGCT			
	GAPDH	F	ATGACTCTACCCACGGCAAG	58	67	NM_017008.4
		R	GGAAGATGGTGATGGGTTTC			
Mouse	ACC1	F	TGGGCACAGACCGTGGTAGT	61	123	NM_133360.2
		R	GCCTGCTGGATTATCTTGGC			
	FAS	F	AAGTTGCCCGAGTCAGAGAACC	65	82	NM_007988.3
		R	ATCCATAGAGCCCAGCCTTCCATC			
	TNF-α	F	GGAACTGGCAGAAGAGGCACTC	64	65	NM_013693.3
		R	GCAGGAATGAGAAGAGGCTGAGAC			
	GAPDH	F	CAGCAATGCATCCTGCACC	64	76	NM_001289726.1
		R	TGGACTGTGGTCATGAGCCC			

Brain-derived neurotrophic factor (BDNF); Ciliary neurotrophic factor (CNTF); Acetyl CoA carboxylase-1; Fatty acid synthetase (FAS); Tumor necrosis factor-α (TNF-α); Glyceraldehyde-3-phosphate dehydrogenase (GAPDH) (GAPDH).

**Table 2:** Isoflavonoid contents in 70% ethanol extract of cooked soybean and chungkookjang made with *B. amyloliquefaciens.* (unit: μg/g dry soybean or chungkookjang).

Ingredients	CSB	BA730	BA731
Daidzin	469 ± 34 ^a^	154 ± 25 ^b^	87.6 ± 13 ^c^
Glycitin	197 ± 22 ^a^	32.7 ± 2.9 ^c^	61.4 ± 7.1 ^b^
Genistin	344 ± 26 ^a^	180 ± 1.4 ^b^	108 ± 13 ^c^
Malonyl daidzin	125 ± 11.4 ^a^	47.3 ± 3.8 ^b^	50.3 ± 5.4 ^b^
Malonyl genistin	150 ± 13.7 ^a^	73.2 ± 6.3 ^b^	81.6 ± 6.8 ^b^
Acetyl daidzin	103 ± 1.3 ^a^	15.2 ± 1.7 ^b^	12.9 ± 1.7 ^b^
Acetyl glyctin	60.2 ± 5.8 ^a^	1.24 ± 0.23 ^b^	1.06 ± 1.21 ^b^
Acetyl genistin	151 ± 11.5 ^a^	0.25 ± 0.01 ^b^	0.16 ± 0.02 ^b^
Glycitein	12.6 ± 1.0 ^b^	19.6 ± 2.1 ^a^	22.5 ± 2.5 ^a^
Daidzein	25.0 ± 2.7 ^c^	1085 ± 101 ^b^	1352 ± 124 ^a^
Genistein	5.9 ± 0.7 ^c^	12.8 ± 1.5 ^b^	25.5 ± 2.1 ^a^
Isoflavonoid aglycones	43.5 ± 4.9 ^c^	1117 ± 123 ^b^	1400 ± 159 ^a^
Total isoflavonoids	2123 ± 198 ^a^	1671 ± 161 ^b^	1803 ± 173 ^b^

CSB, cooked soybean; BA730, chungkookjang made with *B.* *amyloliquefaciens* SRCM100730; BA731, chungkookjang made with *B. amyloliquefaciens* SRCM100731. Values are mean ± SD (*n* = 3). ^a,b,c^ Different superscript letters in values the indicate significant differences among the groups by Tukey’s test at *p* < 0.05.

**Table 3:** Contents of free amino acids in water extracts of cooked soybeans and chungkookjangs made with *B. amyloliquefaciens.* (unit: mg/g dry soybean or chungkookjang).

Ingredients	CSB	BA730	BA731	Ingredients	CSB	BA730	BA731
Phosphoserine	-	125.1 ± 0.14	128.4 ± 0.30	Leucine	0.84 ± 0.07 ^c^	22.2 ± 0.44 ^b^	42.0 ± 0.28 ^a^
Urea	-	-	232.9 ± 0.28	Tyrosine	1.06 ± 0.09 ^c^	28.3 ± 0.12 ^b^	58.1 ± 0.33 ^a^
Aspartic acid	0.55 ± 0.12 ^b^	5.20 ± 0.28 ^b^	6.02 ± 0.42 ^a^	Phenylalanine	1.33 ± 0.10 ^b^	43.4 ± 0.28 ^a^	-
Threonine	0.22 ± 0.05 ^c^	4.26 ± 0.34 ^b^	7.75 ± 0.14 ^a^	α-aminobutyric acid	-	3.68 ± 0.13 ^b^	6.97 ± 0.21 ^a^
Glutamic acid	1.46 ± 0.21	-	-	β-aminoisobutyric acid	-	20.4 ± 0.05 ^b^	36.3 ± 0.24 ^a^
α-aminoadipic acid	-	65.8 ± 0.29 ^b^	78.8 ± 0.12 ^a^	γ-Aminobutyric acid	1.33 ± 0.21 ^c^	10.2 ± 0.14 ^b^	28.7 ± 0.21 ^a^
Glycine	0.61 ± 0.09 ^c^	10.7 ± 0.35 ^b^	20.2 ± 0.16 ^a^	Ethanolamine	0.10 ± 0.03 ^c^	2.47 ± 0.09 ^b^	11.8 ± 0.15 ^a^
Alanine	1.59 ± 0.11 ^c^	19.3 ± 0.19 ^b^	29.8 ± 0.22 ^a^	Ammonia	1.60 ± 0.05 ^c^	12.2 ± 0.08 ^b^	20.3 ± 0.09 ^a^
β-Alanine	1.22 ± 0.14 ^c^	29.7 ± 0.11 ^b^	47.6 ± 0.29 ^a^	Ornithine	-	1.51 ± 0.15	1.51 ± 0.14
Citrulline	-	13.2 ± 0.24 ^b^	20.2 ± 0.16 ^a^	Lysine	0.68 ± 0.02	0.54 ± 0.08	0.99 ± 0.05
Valine	2.17 ± 0.24 ^c^	12.7 ± 0.27 ^b^	24.4 ± 0.21 ^a^	1-methylhistidine	0.27 ± 0.09 ^c^	10.0 ± 0.18 ^b^	14.6 ± 0.16 ^a^
Cysteine	-	40.0 ± 0.11 ^b^	53.3 ± 0.14 ^a^	Histidine	0.27 ± 0.10 ^b^	10.1 ± 0.14 ^a^	9.01 ± 0.27 ^a^
Methionine	0.93 ± 0.08 ^a^	19.6 ± 0.13 ^b^	28.3 ± 0.33 ^a^	Asparagine	-	-	22.8 ± 0.35
Isoleucine	0.86 ± 0.15 ^c^	6.56 ± 0.14 ^b^	15.1 ± 0.10 ^a^	Arginine	3.53 ± 0.14	27.3 ± 0.21 ^a^	23.2 ± 0.22 ^b^

CSB, cooked soybeans; BA730, chungkookjang made with *B. amyloliquefaciens* SRCM100730; BA731, chungkookjang made with *B. amyloliquefaciens* SRCM100731. Values are mean ± SD (*n* = 3). ^a,b,c^ Different letters beside mean ± SD indicate significant differences among the groups by Tukey’s test at *p* < 0.05.

**Table 4:** γ-PGA contents and survival rates (%) of bacteria in chungkookjangs under different pH and bile salts.

Identification	γ-PGA (cm)	pH (%)		Bile Salt (%)	
		7.0	2.0	Oxgall 0.3%	Oxgall 0.6%
CSB	0 ^b^	-	-	-	-
BA730	27.0 ± 3.0 ^a^	100	1.4 ± 0.94 ^b^	35.0 ± 0.85 ^a^	58.0 ± 1.33 ^a^
BA731	30.0 ± 2.0 ^a^	100	6.9 ± 1.21 ^a^	20.2 ± 1.02 ^b^	18.8 ± 0.91 ^b^

CSB, cooked soybeans; BA730, chungkookjang made with *B. amyloliquefaciens* SRCM100730; BA731, chungkookjang made with *B. amyloliquefaciens* SRCM100731. Values are mean ± SD (*n* = 3). ^a,b^ Different letters beside mean ± SD indicate significant differences among the groups by Tukey’s test at *p* < 0.05.
